# From rehabilitation to recovery: protocol for a randomised controlled trial evaluating a goal-based intervention to reduce depression and facilitate participation post-stroke

**DOI:** 10.1186/1471-2377-11-73

**Published:** 2011-06-18

**Authors:** Christine Graven, Kim Brock, Keith Hill, David Ames, Susan Cotton, Lynette Joubert

**Affiliations:** 1 School of Health Sciences, The University of Melbourne, Parkville, Victoria 3052, Australia; 2 Physiotherapy Department, St.Vincent's Hospital Melbourne, PO Box 2900, Fitzroy, Victoria 3065, Australia; 3 Faculty of Health Sciences, La Trobe University and Northern Health, Bundoora, Victoria 3086, Australia; 4 National Ageing Research Institute, PO Box 2127, Royal Melbourne Hospital, Victoria 3050, Australia; 5 Orygen Youth Health Research Centre, Locked Bag 10, Parkville, Victoria 3052, Australia

## Abstract

**Background:**

There is much discourse in healthcare about the importance of client-centred rehabilitation, however in the realm of community-based therapy post-stroke there has been little investigation into the efficacy of goal-directed practice that reflects patients' valued activities. In addition, the effect of active involvement of carers in such a rehabilitation process and their subsequent contribution to functional and emotional recovery post-stroke is unclear. In community based rehabilitation, interventions based on patients' perceived needs may be more likely to alter such outcomes. In this paper, we describe the methodology of a randomised controlled trial of an integrated approach to facilitating patient goal achievement in the first year post-stroke. The effectiveness of this intervention in reducing the severity of post-stroke depression, improving participation status and health-related quality of life is examined. The impact on carers is also examined.

**Methods/Design:**

Patients (and their primary carers, if available) are randomly allocated to an intervention or control arm of the study. The intervention is multimodal and aims to screen for adverse stroke sequelae and address ways to enhance participation in patient-valued activities. Intervention methods include: telephone contacts, written information provision, home visitation, and contact with treating health professionals, with further relevant health service referrals as required. The control involves treatment as usual, as determined by inpatient and community rehabilitation treating teams. Formal blinded assessments are conducted at discharge from inpatient rehabilitation, and at six and twelve months post-stroke. The primary outcome is depression. Secondary outcome measures include participation and activity status, health-related quality of life, and self-efficacy.

**Discussion:**

The results of this trial will assist with the development of a model for community-based rehabilitation management for stroke patients and their carers, with emphasis on goal-directed practice to enhance home and community participation status. Facilitation of participation in valued activities may be effective in reducing the incidence or severity of post-stroke depression, as well as enhancing the individual's perception of their health-related quality of life. The engagement of carers in the rehabilitation process will enable review of the influence of the broader social context on recovery.

**Trial registration:**

Australia and New Zealand Clinical Trials Register (ANZCTR): ACTRN12608000042347

## Background

Inroads in the efficacy of acute stroke management have resulted in reduced mortality rates and morbidity from the event of stroke. However, stroke remains a leading cause of death, and the rise in life expectancy of the Australian population (with the proportion of the population aged over 65 years having considerably increased) has resulted in a higher prevalence of stroke, with an estimated 60 000 events occurring per year [1,2]. About 320 000 people (aged between 16-85 years) had suffered a stroke in 2007 in Australia, with a large subset (42.5%) reporting a disability that resulted in ongoing activity restriction [2]. Disability caused by stroke has enduring social and economic consequences, in part due to the burden on the healthcare system and the dependency on carers for physical and financial assistance [3]. It is important to continue to address the management of the sequelae of stroke, not just in the acute and immediate sub-acute settings, but into the longer-term chronic phase. There is a need to determine and develop strategies that may facilitate longer term outcomes and maximise an individual's capabilities.

Early post-stroke rehabilitation tends to focus on the amelioration of impairments and activity restrictions, so that basic mobility and self-care tasks can be achieved. Upon returning to the community, further sub-acute rehabilitation resources may be required to maximise recovery and prevent deterioration [4,5]. Surprisingly, research on the most effective way to further assist the patient and their carers to re-establish their lives in the community during the first year after stroke, especially with regard to the resumption of valued activities, is scant. Most people who have had a stroke display ongoing activity limitations (such as problems with basic self-care tasks and mobility), and also a resultant negative impact on domestic and community-based activities [3]. There can also be alterations in participation levels, health-related quality of life (HRQoL), and mood status. Variables such as these may reflect the concept of adaptive functioning, and can provide an important gauge of an individual's perception of their current situation [6].

Alteration in mood status, particularly in the form of emerging depression, is a common problem post-stroke. It is estimated that a third of people who have had a stroke will display post-stroke depression (PSD) [7]. Depression is characterised by persistent low mood (of a duration greater than two weeks), often with addition symptoms of: alterations to weight or appetite; disturbed sleep patterns; loss of energy; sense of worthlessness; suicidal ideation; anhedonia; psychomotor retardation and/or agitation. PSD has not only been associated with a diminished re-uptake of previously valued activities, but a reduced perception of HRQoL [8,9]. Thus, there is the need for interventions aimed at not only improving physical impairment and disability, but also addressing mood status, participation, and HRQoL [8]. This was highlighted in a recent systematic review which found that rehabilitation therapies, conducted in patients' community context, which endeavoured to frame the intervention to target patients' valued activities (such as leisure pursuits), showed moderate evidence for improvement in global participation measures and HRQoL [10].

Participation status can also be markedly affected post-stroke, with mobility problems and the presence of depressive symptoms being particularly predictive of adversely altering participation levels [11,12]. Participation, in this context, relates to a person's ability to engage in life situations [13]. This concept also encompasses aspects of personal and social re-integration, and a return to valued and meaningful activities [14]. Valued activities are an individual phenomenon, and may relate to life aspects such as: domestic, family, and social roles; employment; hobbies, socialising; and sporting pursuits [15]. Resumption of previously valued activities may also have an impact on an individual's sense of life quality [8,15]. The perception of HRQoL is generally reported to be reduced post-stroke [16,17]. The concept of HRQoL tries to capture how an individual views the impact of their health status on their quality of life, especially in relation to physical, cognitive, and emotional factors. Presence of depressed mood, low functional activity status, and lack of social support are predictors of HRQoL post-stroke [18,19]. However, a study by Sturm et al. (2004) ascertained that a significant number of people who have had a stroke report a diminished HRQoL despite an overall good functional recovery [8].

Central to the aim of the post-stroke rehabilitation process should be a client-centred framework that adopts a goal-setting model. Meaningful, collaborative goal-setting is an acknowledged essential part of rehabilitation practice [14]. Endorsement of goal-directed behaviour (by the patient, support network, and rehabilitation services) is an important element for ongoing recovery and overall adjustment to post-stroke abilities [20]. In addition, the process of goal setting may assist to reduce carer anxiety and offer active problem-solving coping strategies [21]. The need to provide long-term care for the person who has had a stroke can place carers under considerable emotional, financial, and physical stress [22]. To date, many interventions aimed at reducing the negative impacts of caring have shown minimal effect, and there is a requirement for a heightened understanding of the relationships between patient and carer characteristics, and overall carer outcomes [23]. Awareness of the impact of the broader social context, and in particular consideration of psychosocial factors (such as carer involvement and their emotional well-being) on rehabilitation outcomes is required. Carers can facilitate the recovery process, and their inclusion in the overall rehabilitation model is essential. In shifting to a rehabilitation paradigm that is consumer and carer-directed, there may also emerge a shift from a model based on clinician-driven rehabilitation programs to one that primarily aims to achieve patient-centred recovery.

The issues that are faced by the person who has had a stroke, and their family and carers, in the first year post-stroke are multifactorial and complex. The relationship that has been established in the literature between mood status, participation level, and HRQoL post-stroke warrants further investigation. The exploration of an integrated, goal-oriented approach (utilising activities that the patient nominates as important to their notion of recovery) is the basis of the intervention framework in this randomised controlled trial. The additional effect of such an intervention on the primary carer will also be investigated.

The objectives of the study are to:

(i). investigate the effectiveness of a client-centred, integrated approach to facilitating goal achievement and recovery in the first year post-stroke.

(ii). determine the effect of the integrated approach on carer outcomes.

This paper provides a detailed description of the methodology of this post-stroke interventional study, based on the following hypotheses:

- The primary hypothesis for the participants who have had a stroke is that an integrated approach to facilitating goal achievement in the first year after stroke will result in less depressed mood, as measured by the Geriatric Depression Scale (15 item) [24,25].

- For the carer participants, the primary hypothesis is that the integrated approach will result in an enhanced HRQoL, as assessed by the Assessment of Quality of Life (AQoL) [26].

## Method/Design

This is a single blind randomised controlled trial of a multifaceted, client-centred intervention for post-stroke management.

### Participants

All patients admitted to the inpatient hospital rehabilitation unit with the primary diagnosis of acute cerebrovascular accident (inclusive of cerebral infarction, intracranial haemorrhage, and subarachnoid haemorrhage) will be eligible to participate in this study provided informed written consent is obtained. Participants will be recruited from two rehabilitation units of a tertiary teaching hospital in Melbourne, Australia. Exclusion criteria are: primary cause of disabilities is a diagnosis other than stroke; associated head trauma (such as fracture); epidural and subdural haemorrhage; cerebrovascular event due to the presence of malignancy; discharge destination is to a high level care facility; rehabilitation inpatient length of stay of less than 4 days duration; and participant resides greater that one hour travel from the rehabilitation unit.

Patients with communicative and cognitive deficits will be eligible to be recruited to the study, provided a 'person responsible' grants written consent for participation. It is acknowledged that these participants may not be able to participate in all assessment components. However, exclusion of these participants would limit the generalisability of the trial, and also exclude potentially valuable information acquired from their carers. Patients with cognitive and communication deficits are also often significant consumers of healthcare resources [27]. Patients who are from culturally and linguistically diverse communities will also be eligible to participate in this study, with the assistance of interpreters.

Primary informal carers will be invited to participate in this study if it is envisaged that they will provide at least five hours per week assistance to the patient participant with personal, domestic, or community activities of daily living. Care services provided by formal agencies, trained professionals, or paid carers will not be considered in this trial.

### Procedures

**Figure 1:**
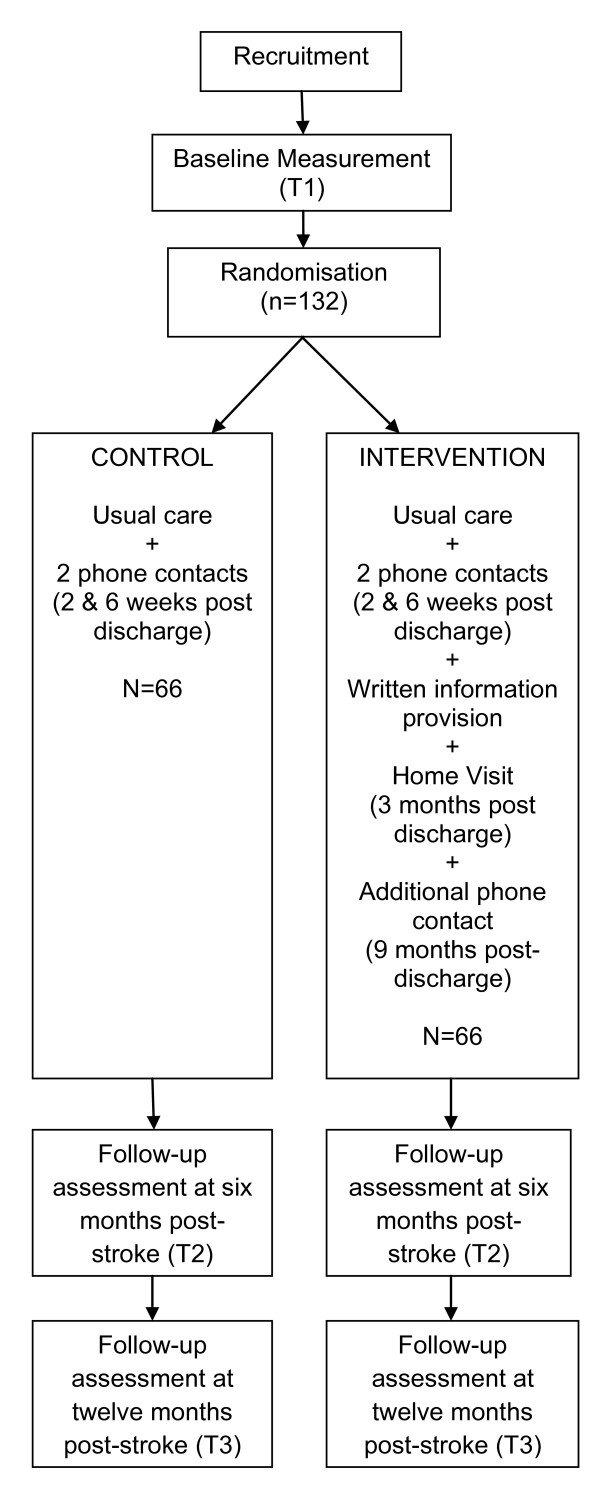
Study design - randomised controlled trial

The design of this study is depicted in Figure 1. Patient participants will be formally assessed at three timepoints (T1 = rehabilitation discharge, T2 = six months post-stroke, and T3 = twelve months post-stroke). The baseline assessment will be conducted by an experienced allied health clinician blind to group allocation. This assessment (T1) will occur after obtaining written informed consent, but prior to randomisation, using the following measures:

- cognition, using the Mini-Mental State Examination (MMSE) [28]. The MMSE provides a screen for cognitive impairment based on a 30 point questionnaire. It assesses various cognitive functions including: orientation, registration, attention and calculation, recall, language, and visual-spatial ability. A score of ≤ 23 points can provide preliminary evidence of cognitive impairment [29].

- activity/functional status, using the Functional Independence Measure - motor component (FIM-motor) [30]. The FIM assesses the amount of assistance that a person requires when performing basic activities of daily living (on a 7 point scale). It has both motor and cognitive subscales, however, only the motor components (13 items) will be scored in this study.

- mood status, using the Geriatric Depression Scale - 15 items (GDS-15) [24,25]. The GDS-15 is a screening tool for depressed mood status. Fifteen questions are scored based on 'yes/no' answers. Adequate reliability, validity, and sensitivity parameters have been determined when the GDS has been applied to stroke populations [31].

- self-efficacy, using the Strategies Used by People to Promote Health questionnaire (SUPPH) [32]. The SUPPH rates the degree of confidence that a person has in conducting specific self-care behaviours. Each item is rated on a 5 point scale of confidence, with higher scores indicating greater self-care self-efficacy. The 23 item SUPPH will be used in this study, as the items have been modified for use with people who have had a stroke [33]. Three main subscales are assessed: coping, reducing stress, and enjoying life.

In addition, general demographic information will also be recorded at the T1 assessment, including: participant's age, side/site/type of stroke, past medical history and co-morbidities, history of depression, living arrangements, and availability of an informal carer.

Collaborative goal setting will be conducted at discharge by the treating inpatient rehabilitation team members using Goal Attainment Scaling (GAS) [34] for all participants. Inpatient rehabilitation staff will undergo training regarding goal setting principles and the application of GAS (inservice program, booklet, and competency questionnaire). Rehabilitation team members will specifically discuss with the patient (and carer, if available) what activities they participated in prior to their stroke and which of those activities could represent goals to be pursued in the first year post-stroke. The determination of relevant, new activities based on current activity status will also be considered. Goals can be set across the domains of body structure/function, activity status, and participation status, depending on the ability level of the individual. However, emphasis will be placed on setting goals that enhance home and community participation levels, and reflect valued activities according to the patients' preferences. Should the goals that are nominated by the patient be considered unrealistic by the rehabilitation team, efforts will be made to determine achievable, preliminary goals that provide the foundation for more complex goals. This will be done via discussions with the patient until a collaborative compromise is reached.

An assessor, who is blind to group allocation, will conduct interviews in the participants' place of residence at T2 (six months post-stroke) and T3 (twelve months post-stroke). The assessor will repeat the measures taken at T1 (GDS-15, MMSE, FIM-motor, SUPPH), and will also include the additional outcome measures of:

- participation, using the (1) the Activity Card Sort (ACS) [35,36] and (2) the London Handicap Scale (LHS) [37]. (1) The ACS aims to measure the impact of disability on participation status by quantifying the percentage level of retained and lost activities. Q-sort methodology is employed using photo cards that depict everyday activities over three occupational performance domains (household, social/educational, and leisure). The ACS-AUS (Recovery version) will be utilised in this study, which consists of 82 activities validated to the Australian population [36]. (2) The LHS aims to measure the level of participation restriction across the six dimensions of the WHO disability framework (mobility, physical independence, occupation, social integration, orientation, and financial self-sufficiency) 

- health-related quality of life, using the Assessment of Quality of Life (AQoL) instrument [26]. The AQoL measures five dimensions of Health Related Quality of Life (HRQoL): illness, independent living, social relationships, physical senses, and psychological well-being. The tool consists of 15 items, each with four response levels. The AQoL has shown to be sensitive to changes in health states, and Australian population norms are available [39].

There will also be documentation of which health and community services are utilised by the participants who have had a stroke at each assessment timepoint during the first year post-stroke.

The primary outcome measure for the participants with stroke is depressed mood, which will be assessed by the GDS-15. Secondary outcomes for the participants with stroke are: participation (ACS and LHS); HRQoL (AQoL), activity/functional status (FIM-motor); self-efficacy (SUPPH); and cognition (MMSE).

Carer outcomes will be collected at T2 and T3, which include the GDS-15, ACS, and AQoL, and the Zarit Burden Index (ZBI) [40]. The ZBI aims to measure carers' perception of their caring role, and the degree to which they feel burdened by that role. Twenty-two items are measured on a 5 point scale, and an overall burden score is formed (0 = no burden to 88 = severe burden). Concepts within the scale cover notions of personal life, social life, health, emotional well-being, and finances [40].

The Human Research Ethics Committees (HREC) of both St.Vincent's Hospital Melbourne and The University of Melbourne have granted approval for this project. Written informed consent will be required from patients and their informal carer. If a patient is unable to grant informed consent (for example, due to cognitive deficits, dysphasia, reduced level of alertness), then a 'person responsible' (as defined in HREC guidelines) is eligible to give consent.

### Randomisation

Participants will be allocated to the control or intervention group of the study by a member of the research team not involved in the assessment process, using a computer-generated random allocation sequence (in block sizes of six) - with further stratification based on the participant's admission FIM-motor score. Participants with an admission FIM-motor score of less than or equal to 46 will be stratified into a 'severe' group, whilst those patients who have a score of greater than 46 will be stratified into a 'mild' group [41]. Participants (patients and their carers) are blind as to which group they have been allocated. In the Patient Information and Consent form, all participants are informed that they will be contacted at two and six weeks post-discharge from inpatient rehabilitation. It is also outlined that the participants may be offered further opportunities to discuss their progress and receive additional therapy and/or community resources. Permission was sought and granted for limited disclosure regarding the two interventions from the HREC. Sealed opaque envelopes are used to conceal the allocation, and participants will be assigned in order of completed baseline data set obtained by an assessor blind to group allocation.

### Interventions

#### Intervention group

Participants in the intervention group will be provided with a multi-factorial, integrated approach which incorporates both standardised and responsive components. At the point of discharge from inpatient rehabilitation, the participants will receive:

1) written material developed by the National Stroke Foundation relating to recovery after the event of a stroke (Booklet: *Long Term Recovery*, and seven Factsheets: *Movement and exercise after stroke, Depression after stroke, Medication after stroke, Communication after stroke, Sexuality after stroke, Diet after stroke, Thinking and perception after stroke*) [42].

2) written stroke information resources, including contact phone numbers (and websites, if available) for: The National Stroke Foundation, The Stroke Association of Victoria, Brain Foundation of Victoria, Headway Victoria, the participant's local Municipal Council, and a local stroke support group (if available). The participants will also receive the contact details of the inpatient Rehabilitation Unit and the project research co-ordinator. Carer participants will be given the contact details of: Carers Victoria (including the Carer Counselling Service, and Commonwealth Carer Resource Centre Victoria), Commonwealth Carer Respite Centre, and local carer support groups (if available).

3) a copy of the goals that were collaborately devised by the participant and the rehabilitation team during the final two weeks of the inpatient rehabilitation admission

In addition to the above interventions,

4) written correspondence will be sent (at the point of inpatient rehabilitation discharge) to the General Practitioner outlining the participant's involvement in the study, the aim of the study, and a copy of the collaborative goals.

5) written correspondence will also be sent to the main community-based rehabilitation services that the patient has been referred to for ongoing management (such as Community Rehabilitation Centre, Rehabilitation in the Home or other domiciliary-based services).

6) phone contact will be made with participants at two and six weeks post-discharge. The nature of these contacts is to enquire about:

- current participant activity status (for example, *How are you currently managing being at home? How are you managing with your everyday activities? *Verify which post-discharge services are in place).

- how the participant is progressing with their goals (including identification of barriers, and discussion regarding possible solutions).

- any falls, accidents, medical issues arising, or injuries sustained.

- mood status (for example, *How are you feeling? Is there anything that is worrying you? Are there any concerns or needs that you feel are currently not being addressed?*).

- presence or absence of informal supports (such as family, friends, and community-based supports). Determine what type of support is being offered (for example, emotional support and/or practical support).

- whether the participant is interested in attending a stroke support group (if one is available in the local area).

7) Home visit to participant's residence at three months post-discharge. The enquiries made during this visit follow a similar format as the previous phone contacts. It is considered by the researchers that this informal 'face-to-face' visitation is a key component, as it takes place in the patient's context. This may allow for a better overview and evaluation of the patient's abilities through actual visualisation of their current activity status. In addition, environmental factors (within the home and immediate community) and carer/family interactions can be gauged. During the home visit, emphasis will also given to the 'best effort' that the participant has achieved to date regarding their activity and participation status. Verbal encouragement will be given to maintain or increase the frequency or achievement level of their activity status, as appropriate. At this three month timepoint, enquiries will also be made as to the patient's perception of the utility of the written material that was received at discharge (Booklet and Factsheets). The participants will be asked whether they have read the written material, and whether or not they considered the information to have been useful.

8) Interventions determined on a 'needs' basis, to facilitate goal achievement and community re-integration.

9) Review of assessment findings at 6 months, and implement interventions in response to the obtained data.

10) Telephone contact at nine months post-stroke to re-evaluate status and initiate any additional interventions, as required.

All the standardised intervention components (listed above as numbers 1-10) will be conducted by the primary researcher, who is a senior physiotherapist experienced in neurological rehabilitation, and not involved in the assessments. Should additional interventions be required, referrals will be made to relevant healthcare resources with correspondence outlining the arising issue and a request for appropriate assessment and management (for example: assessment of community access options with consideration of utilising an electric scooter to enable greater outdoor distances; review of urinary incontinence by the catchment Continence clinic; request for the general practitioner to assess and manage a patient's evolving low mood).

Verbal support will be given to the participants and their carers at all contacts, including acknowledgement of achievements to date and encouragement to continue to pursue activities and goals. Verbal support will also particularly be offered to those participants who display evidence of low mood status. Intervention group participants will continue to receive usual care from health and community resources as deemed appropriate by their medical consultants and treating team. The telephone details for the researcher will be given to the participant at each contact point, with the recommendation to make contact with the researcher if they have any queries or concerns about their recovery post-stroke.

During the phone contacts and home visit, issues or 'flags' may emerge that give rise to the need for additional interventions. Table 1 outlines the guidelines for interventions in response to certain 'flags'. However, as the interventions are based on the arising needs of an individual participant, the interventions listed in Table 1 are not exhaustive, but rather a strategy for initial clinical decision making. 'Flags' may also be discerned during scrutiny of the outcome data that is collected at the blind assessments conducted at six months post-stroke. Some of the intervention options may not need to be exercised, as the current treating team may already be aware of the issues and be providing the relevant service.

**Table 1:** Guidelines for interventions based on arising 'flags'

Domain	'Flags'	Intervention options
Post-discharge services	- The services organised at discharge from inpatient rehabilitation have not commenced as scheduled	- Contact relevant service to determine referral status - Liaise with inpatient rehabilitation clinicians to verify referral status

Activity status	- Decline in activity status/functional decline (including PADL, mobility, continence) - Failure to progress in activity status in valued activities	- If participant is currently attending community-based rehabilitation services, liaise with relevant team members (such as OT/PT/SP) - If participant is not attending any community-based rehabilitation services, refer to relevant health professional for assessment and management - Inform participant about local services (such as exercise groups, hydrotherapy) as appropriate - Refer to GP for review (to exclude medical basis for decline in functional ability) - Refer to Continence Clinic, if appropriate - Refer to ACAS, if appropriate

Cognition	- Decline in cognitive function (reports from patient, family, carer) - Safety concerns due to cognitive impairments - Evidence of marked change in MMSE performance between assessment timepoints	- Refer for medical evaluation (such as GP/Rehabilitation Medicine Specialist/Geriatrician). - If participant is currently attending community-based rehabilitation services, liaise with OT regarding cognitive assessment and management - If participant is not attending any community-based rehabilitation services, refer to OT for assessment and management - Referral to Cognitive Dementia and Memory Service as appropriate

Falls	- Episodes of falls - Fear of falling limiting function	- Monitor number and nature of falls during contacts with participant. - If participant is currently attending community-based rehabilitation services, liaise with relevant team members. If team is unaware of falls, request a falls risk assessment. - If participant is not attending any community-based rehabilitation services, refer to relevant health professional for a falls risk assessment and management

Mood status	- GDS-15 score of ≥ 6 points, or marked change in GDS-15 score between assessment timepoints - Evidence during contacts of depression or mood change	- Referral to GP - Encouragement to participate in valued activities - Encouragement to participate in physical activity (as able) and enhance social contacts - Referral to CATS if urgent assessment required

Goals/Participation status	- Failure to resume, or reduced participation in, valued activities that should be achievable post-stroke - goals not being achieved based on GAS ratings at 6 & 12 month assessments	- identify barriers to goal achievement - re-establish goals as required (with regard to both timeframes and attainment level) - If participant is currently attending community-based rehabilitation services, liaise with relevant team members (such as OT/PT/SP) - If participant is not attending any community-based rehabilitation services, refer to relevant health professional who can assist with facilitating and enhancement of participation status and goal attainment

Health/Medical status	- hospital inpatient re-admission during the 12 month follow-up period	- if the researcher has knowledge of the admission, contact by phone at two weeks post-discharge to monitor status.

Informal support	- absence of informal supports that is resulting in evidence of loneliness or lack of emotional support	- Provide information to the participant about relevant local community groups/services. Facilitate referral to group/service - Provide information about closest Stroke Support Group

Carer status (for consented carer participants)	- Evidence of reduced carer coping or stress during contacts - GDS-15 score of ≥ 6 points - Zarit Burden Interview > 24 points	- Aim to identify causes of reduced coping/stress - Provide information regarding carer resources (refer to information given at inpatient rehabilitation discharge timepoint). Discuss options. - If the patient participant is currently attending community-based rehabilitation services, liaise with relevant team members (such as SW) - Encourage GP review - Offer ongoing verbal support and encouragement to the carer in their role during contacts - Continue to engage carer in the rehabilitation process

PADL: Personal activities of daily living

OT: Occupational therapist

PT: Physiotherapist

SP: Speech Pathologist

GP: General practitioner

ACAS: Aged Care Assessment Service

MMSE: Mini-mental State Examination

GDS-15: Geriatric Depression Scale (15 item)

CATS: Crisis Assessment and Treatment Service

GAS: Goal Attainment Scaling

SW: Social Worker

#### Control group

Participants in the control group will receive usual care as arranged by the treating team at the point of discharge from the inpatient rehabilitation admission. In addition, control group participants will be contacted at two and six weeks post-discharge by an experienced allied-health clinician. This study design is implemented to ensure that the participants remain blind to group allocation. No interventions or advice will be offered to the control group participants by the researchers at these timepoints. A general enquiry will be made as to whether the services that were arranged at discharge have commenced. The control group participants will only receive an intervention by the researchers if there are particular indicators of risk, such as verbal ideations of suicide/self-harm or a GDS-15 score ≥ 14.

### Power and sample size calculations

Power calculations for this study are based on nine month post stroke data (obtained six months post-intervention) for GDS-15, from a study performed by Lai et al. (2006) [43]. Based on a power of 80% and the criterion for significance set at 0.05 (two-sided), the proposed sample size is 55 participants for each arm of the study. This computation assumes that the mean difference between groups is -1.4 (corresponding to means of 2.0 versus 3.4) and the common within-group standard deviation is 2.6 (based on SD estimates of 1.8 and 3.2). Allowing for a 20% drop-out rate, the aim is to recruit 66 participants in each group, giving a total sample of 132 participants.

### Statistical methods

The primary outcome measure for the participants who have had a stroke is the total score on the GDS-15 measured at 12 months post-stroke, with scores ranging from 0 to 15 (with 15 indicating more severe symptoms). The main independent variable is group, which comprises two levels: intervention and control. When considering group differences on the GDS-15 at 12 months, it is important to control for severity of disability (initial FIM-motor score), previous history of depression (yes/no), length of stay in hospital, and discharge GDS-15. Mixed regression models for repeated measures will be used to examine group differences over time on the GDS-15 controlling for these variables. These models will also be used to assess group differences on patient secondary outcomes, and group differences on carers' variables.

## Discussion

The recommendation for healthcare clinicians to incorporate a client-centred approach to the rehabilitation process is well entrenched in guidelines for stroke rehabilitation and current policy trends [44]. However the adoption of such practice is variable, and many goals of stroke rehabilitation remain clinician-directed and focused on basic mobility and self-care activities. There is a distinct need for a wider adoption of patient-determined goals to adequately focus on activities that have intrinsic meaning for the individual. It is hoped that such a shift in focus will produce more favourable outcomes with regards to mood status, participation levels, and perception of health-related quality of life. The additional involvement of carers as 'partners' in the rehabilitation process may also lead to favourable patient outcomes, as well as lessening the emergence of adverse carer outcomes. The goal of this study is to investigate whether offering an integrated intervention to facilitate goal achievement is more effective for these outcomes than usual care.

There are many complexities that arise when performing research on a community-based post-stroke cohort. In this trial there will inevitably be variation between patients with regards to the time from stroke event to baseline data collection (discharge from inpatient rehabilitation). The other formal assessments are taken at a fixed time post-stroke (six and twelve months). The intervention for this study is essentially non-standardised, making description of the protocol challenging. Its operation is based on emerging 'flags' that activate consideration of a relevant intervention to address the identified situation. However, a distinct advantage of such a protocol is that it enables the intervention to be responsive and based on individualised needs. There is evidence to support the adoption of community-based interventions that endeavour to take into account the valued activities of the person who has had a stroke [10]. The effect of the event of a stroke on mood status, participation levels, and health-related quality of life are well documented. The interest should now shift to how to best intervene to positively alter these outcomes, for both the stroke patient and their carer.

If the intervention in this study is shown to be of benefit to both the patient and carer, it would provide the basis for a model of community-based rehabilitation management for people who have had a stroke and their carers, that adopts a robust client-centred approach. This model could conceivably be implemented within the current sub-acute rehabilitation services framework, without need for extensive additional resources.

## Competing interests

The authors declare that they have no competing interests.

## Authors' contributions

KB and LJ conceived and obtained funding for the study. All authors (KB, LJ, KH, DA, SC, and CG) participated in the design of the study and continue to contribute to its ongoing co-ordination. In addition, SC performed the power and sample size estimates, and consulted on the likely statistical methods that will be employed during data analysis. CG drafted the manuscript. All authors critically reviewed and approved the final manuscript.

## Pre-publication history

The pre-publication history for this paper can be accessed here:

http://www.biomedcentral.com/1471-2377/11/73/prepub
